# Racial inequalities in eligibility and access to lung cancer screening: systematic review of United States studies

**DOI:** 10.1186/s12889-025-24761-2

**Published:** 2025-11-17

**Authors:** Ya Yambao Yang, Courtney McNamara, David Stuckler

**Affiliations:** 1 Department of Public Health Sciences, School of Medicine, University of California, 4610 X Street, Davis, Sacramento, CA 95817 USA; 2 Population Health Sciences Institute, University of Newcastle, Newcastle upon Tyne, NE2 4AX UK; 3 Department of Social and Political Sciences and Dondena Research Centre on Population Dynamics, University of Bocconi, Milan, Italy

**Keywords:** Lung cancer screening, Racial inequality, Access, Eligibility

## Abstract

**Introduction:**

In 2021, the United States Preventive Services Task Force introduced new guidelines aimed at reducing racial inequalities by lowering the smoking threshold and expanding age eligibility for screening. However, while some studies suggest these updates have increased eligibility and access, the impact of these changes on racial inequalities in lung cancer screening (LCS) eligibility and access has yet to be systematically reviewed. This study aims to synthesize existing evidence on racial disparities in LCS eligibility and access in the U.S. following the 2021 guideline reform.

**Methods:**

We searched PubMed and Web of Science for studies using keywords related to race, lung cancer, and screening in the United States. The final search was conducted on July 25th, 2024. Articles were included if they quantified access or eligibility for one or more racial groups, enabling quantification of either absolute or relative inequality. Our final analytical sample included 26 articles.

**Results:**

Of the 26 studies reviewed, 12 evaluated disparities in eligibility, and 17 assessed racial disparities in LCS access. All eligibility studies reported that Black Americans, Hispanics, and Asian Americans had lower eligibility rates compared to White Americans; estimated gaps between White and Black Americans ranged from 3.4 to 26.9% points and between 4.7 and 33.1 ppts between Whites and Hispanics. Gaps in access to LCS, conditional on eligibility, were narrower, ranging from 1.42 to 12.9 ppts. Higher disparities were observed on the U.S. East Coast compared to the West Coast.

**Conclusion:**

Despite changes to guidelines aiming to ameliorate inequalities LCS, inequalities in both eligibility and access remain pronounced, especially for Black and Hispanic Americans. Further reforms are needed to adjust for lower eligibility among groups with higher levels of LCS need. Additionally, geographic differences, such as more pronounced disparities on the East Coast, suggest that regionally tailored approaches may support efforts to advance racial equity.

**Supplementary Information:**

The online version contains supplementary material available at 10.1186/s12889-025-24761-2.

## Introduction

Lung cancer is the second most common and the leading cause of cancer deaths in the United States [1]. It disproportionately affects racial and ethnic minorities, particularly Black Americans. According to the American Lung Association, Black men (12%) and women (16%) are significantly less likely than their White counterparts to be diagnosed at an early stage (16% and 20%, respectively) [2]. The survival rate for Black individuals (21%) was also lower than White individuals (25%), partially due to their late stage of diagnosis [2]. Understanding the reasons behind these disparities is crucial for improving health outcomes and reducing lung cancer deaths among minority populations [2].

Despite black populations having considerably elevated needs and potential benefits from lung cancer screening (LCS), they are the least likely to obtain it [3, 4]. This pattern reflects what has been theorized as the ‘inverse care law framework [5], whereby those with the greatest need often receive the least care, thought to reflect market forces in healthcare [6–8]. Although the inverse care law was initially associated with market-driven healthcare systems, it has more recently been applied to explain how structural racism, inequitable policies, and historical exclusion perpetuate disparities in access to care in the United States [6, 7]. Analyses of the US Behavioral Risk Factor Surveillance Survey consistently reveal a stubborn racial gap in both LCS eligibility and access between White Americans and other minority groups [3, 9–11].

Historically, lung cancer screening guidelines were based on clinical trials that predominantly involved White men, which limited their applicability to diverse populations [12, 13]. This bias in the design of clinical trials has been linked to inadequate consideration of racial and ethnic disparities in lung cancer outcomes. As a result, previous screening guidelines did not fully consider the risk factors of racial minorities, such as Black Americans, who tend to be diagnosed at a younger age and with fewer pack-years of smoking. Additionally, some Asian American subgroups, including Korean and Chinese Americans, experience elevated lung cancer incidence among never-smokers, especially for women. Potential contributing factors include genetics, secondhand smoke, cooking fumes, and air pollution. However, these factors are rarely captured in eligibility criteria.

Recognizing the persistent racial inequalities in LCS access, in 2021, the US Preventive Services Task Force (USPSTF) updated their 2013 guidelines to lower the smoking threshold from 30 to 20 pack-years and expanded age eligibility from 55 to 50 [14]. These changes were specifically designed to improve access to screening for minority populations who may not have met the previous criteria. As stated by the USPSTF, “Screening for lung cancer in persons at an earlier age and with fewer pack-years of smoking (i.e., 20 pack-years) may also help partially ameliorate racial disparities in screening eligibility.” [14] The changes were essential because Black individuals are statistically more likely to be diagnosed with cancer at a younger age than their White counterparts due to a combination of genetic factors, socioeconomic disparities, and reduced access to healthcare [15].

Whether these changes have helped ameliorate inequalities is unclear. Several studies found that the changes decreased racial disparity in eligibility and access to LCS for Black Americans and White Americans. However, many of these studies only examined the Black American population, but not other racial minorities [3, 10, 16–18]. While some studies suggest these updates have improved eligibility and access [19–21], there is no comprehensive systematic review to summarize existing evidence on whether changes in screening guidelines have influenced racial disparities in accessing and screening uptake. A recent systematic review analyzed racial differences in adherence to LCS follow-up but did not specifically investigate changes linked to guideline reforms [22].

In the United States, the USPSTF recommends annual lung cancer screening using low-dose computed tomography (LDCT) for adults aged 50 to 80 years who have a 20 pack-year smoking history and currently smoke or have quit within the past 15 years [14]. While these national guidelines were created to standardize access, implementation, and coverage, they vary widely across states and insurance providers [23]. Under the Affordable Care Act, most private insurers and Medicaid expansion programs are required to cover LCS costs for eligible individuals [23, 24]. However, racial and ethnic minorities continue to face systemic barriers that affect eligibility and access [23–25]. For instance, while eligibility criteria have expanded by age and smoking history, it does not account for elevated lung cancer risk from non-smoking-related exposures, such as air pollution, secondhand smoke, or occupational hazards [25]. These exposures are more common in Black Americans and other marginalized communities. Moreover, smoking history is often under-documented in electronic medical records, especially among marginalized populations with inconsistent primary care access [23, 25]. Additionally, even when eligibility is met, access may still be hindered by insurance coverage, referral delays, limited screening sites, and a lack of culturally and linguistically appropriate services [23–25].

Here, we perform a systematic review evaluating racial inequalities in access to and eligibility for LCS. Specifically, we evaluate for which racial group inequalities persisted and whether improvements have been observed in association with 2021 guideline reforms. We additionally investigate both absolute and relative racial inequalities in eligibility for and eventual access to LCS.

## Methods

We performed a systematic review examining racial inequalities in eligibility and access to LCS in the United States following the Preferred Reporting Items for Systematic Reviews and Meta-Analyses (PRISMA) guidelines [26]. Our study was preregistered in PROSPERO (CRD42024579806).

### Search strategy

We searched PubMed and Web of Science on July 25th, 2024 for keywords pertaining race, lung cancer, and screening. To do so, we drew upon previously established search terms for each keyword [27–29]. For race, this involved permutations such as people of color, minority, ethnicity, and immigrant, among others [27]. For lung cancer, it mainly consisted of lung disease and lung neoplasm [28]. For screening, we included low-dose computed tomography [29]. This yielded the following search strings: (“race” OR “people of colo*” OR “minori*” OR “ethnic*” OR “immigrant*” OR “emigrant” OR “emigrat*” OR “African American” OR “African-Americans”) AND (“Lung cancer” OR “lung disease” OR “lung neoplasm”) AND (“Screening” OR “low-dose computed tomography” OR “LDCT”). Appendix 1 provides the verbatim search strings for both databases.

**Fig. 1:**
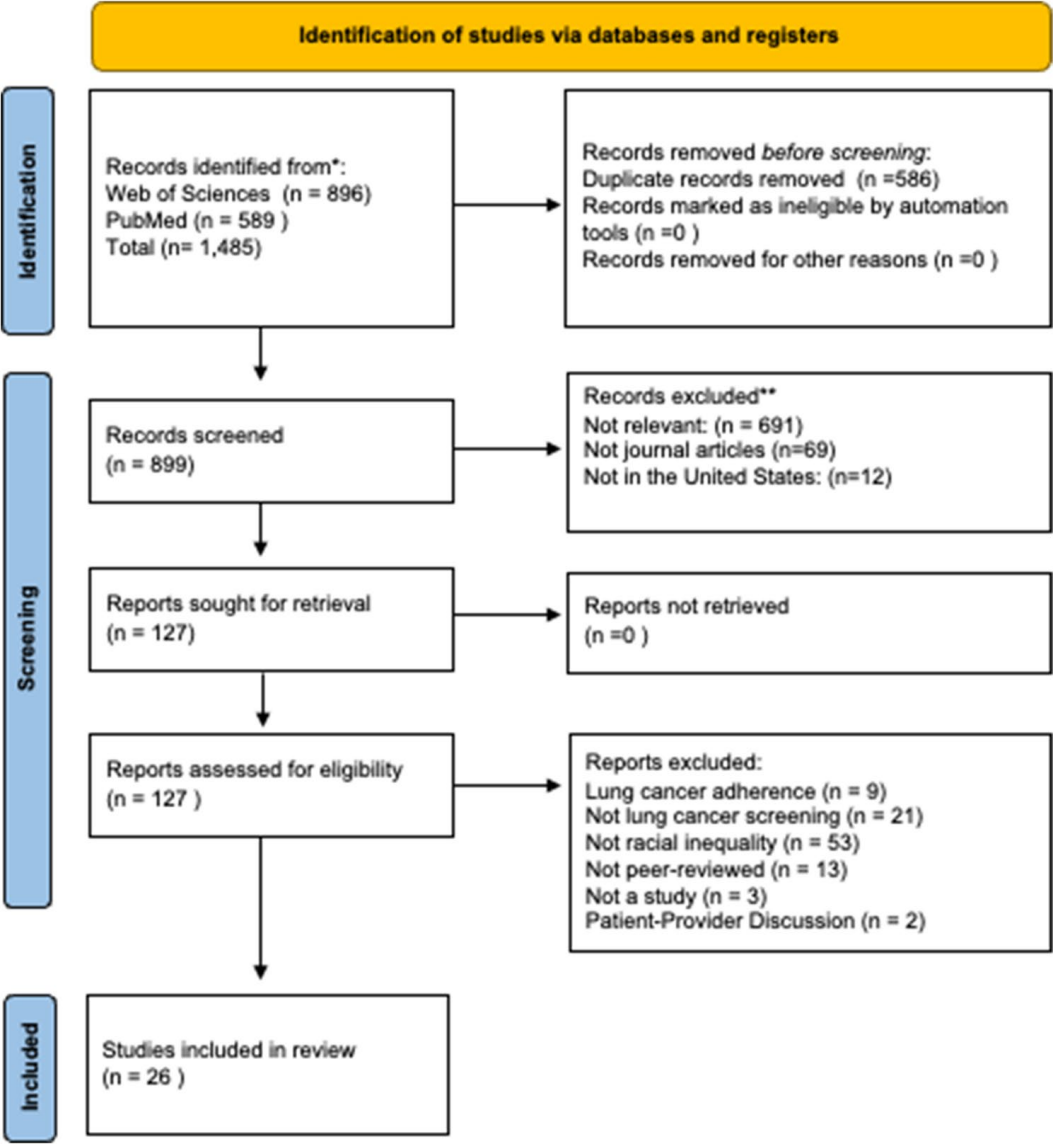
PRISMA Flow Diagram of Study Inclusion

This initial search generated 1,485 articles (PubMed = 589; Web of Science = 896). These articles were imported to Zotero reference management software, where 586 were identified as duplicates. After removing the duplicates, we were left with 899 articles for screening and eligibility. Figure 1 shows the PRISMA flow diagram for study inclusion.

### Inclusion and exclusion criteria

We applied a series of inclusion and exclusion criteria, as follows. Articles were included if they: (i) were based in the United States (ii) evaluated eligibility or access to LCS and (iii) quantified racial inequality. We excluded articles that were not in English or were not peer-reviewed published papers. Furthermore, we excluded articles that examined adherence to LCS or evaluated access to potential determinants of LCS and not access not LCS per se. Two reviewers (YY and DS) independently screened titles, abstracts, and full-text. DS also conducted an additional quality control check by randomly selecting 20% of the articles for re-review. Cases of dispute were resolved by CM.

Applying these criteria, we excluded 12 articles that were not based in the United States, 69 that were not journal articles, and 691 for not evaluating LCS and quantifying racial inequality. This resulted in a total of 127 articles, which we retrieved the full texts for evaluating eligibility. Upon reviewing the complete text, we excluded 9 articles for evaluating lung cancer adherence, 21 for not examining LCS, 53 for not covering racial inequality, 13 for not being peer-reviewed, 3 for not being a study, and 2 for exploring the effect patient-provider discussion had on LCS. Ultimately, our final analytical sample included 26 articles.

### Data extraction and analysis

We extracted study parameters into a summary Excel table capturing: author, location of study data, data source, study design, racial comparison, inequality measure, and access outcome. For inequality measures, we identified whether studies reported absolute or relative inequality. Inequality is defined as measurable differences in lung cancer screening eligibility or access between racial or ethnic groups. Eligibility inequality refers to the amount of individuals who met the USPSTF screening criteria. On the other hand, access inequality refers to the actual utilization of screening services. Inequality was quantified using absolute percentage-point differences or relative measures such as odds ratios. Where possible, we report absolute inequalities as they better enable comparisons over time and across differing racial group comparisons, since relative inequality measures can vary due to changing denominator sizes. Access outcomes were defined as eligibility and/or actual access to screening.

For those studies focusing on eligibility, we also extracted whether they used the 2013 or 2021 U.S. Preventive Services Task Force lung cancer guidelines or both. This was then used to evaluate the potential changes in inequalities in eligibility over time associated with the guideline reforms. Meta-analysis was not appropriate for this review due to substantial heterogeneity across the included studies in terms of study design, population denominators, data sources, and geographic regions. However, we quantitatively compared the magnitude of racial disparities disaggregated by race, region, and access to outcome. We did not report on ‘other’ races as these included heterogeneous categories which were not comparable across studies. 

## Results

### Descriptive characteristics

Table 1 summarizes the 26 included studies and their main characteristics [3, 33, 35, 36]. These studies were mainly based on cohort (*n* = 17) or cross-sectional designs (*n* = 8), with one case-control study. The studies were all conducted, at the time of writing, within the last decade, between 2016 and 2024, although the data covered time periods spanning the years 1993 to 2023. Seven studies used data from the Centers for Disease Control and Prevention’s Behavioral Risk Factor Surveillance System (BRFSS) database [14].

**Table 1:** Racial Inequalities in Eligibility (n=12) and change in estimated racial inequalities between 2013 and 2021 guidelines

**Study**	**Study Population**	**Racial group**	**Eligibility for Lung Cancer Screening**		**Absolute Inequality**		**Change in Inequality between Guidelines**
			**2013**	**2021 **	**2013**	**2021 **	
Reese et al. (2021) [21]	Behavioral Risk Factor Surveillance System	White	31.1%	40.9%	Reference	Reference	Reference
		Black	16.3%	28.8%	14.8%	12.1%	−2.70%
		Hispanic	10.5%	18.7%	20.6%	22.2%	1.60%
Narayan et al. (2021) [30]	Behavioral Risk Factor Surveillance System	White	12.0%	15.0%	Reference	Reference	Reference
		Black	7.0%	9.0%	5.0%	6.0%	1.00%
		Hispanic	4.0%	5.0%	8.0%	10.0%	2.00%
		Asian American	4.0%	5.0%	8.0%	10.0%	2.00%
		American Indian	17.0%	21.0%	- 5.0%	−6.0%	1.00%
Aredo et al. (2022) [31]	Multiethnic Cohort	White	43.5%	49.6%	Reference	Reference	Reference
		Black	27.7%	38.4%	15.8%	11.2%	−4.60%
		Hispanic	28.9%	37.3%	14.6%	12.3%	−2.30%
		Asian	33.4%	40.0%	10.1%	9.6%	−0.50%
		Native Hawaiian	46.3%	56.7%	−2.8%	−7.1%	4.30%
Pinheiro et al. (2022) [19]	REGARDS study	White	27.2%	34.6%	Reference	Reference	Reference
		Black	19.2%	28.8%	8.0%	5.8%	−2.20%
Williams et al. (2022) [32]	Behavioral Risk Factor Surveillance System	White	21.9%	35.8%	Reference	Reference	Reference
		Black	16.0%	28.5%	5.9%	7.3%	1.40%
		Hispanic	9.8%	18.0%	12.1%	17.8%	5.70%
Liu et al. (2023) [33]	Boston Medical Center’s Clinical Data Warehouse	White	60.6%	72.8%	Reference	Reference	Reference
		Black	44.3%	64.7%	16.3%	8.1%	−8.20%
		Hispanic	59.3%	70.4%	1.3%	2.4%	1.10%
Li et al. (2024) [34]	Health and Retirement Study	White	35.1%	63.4%	Reference	Reference	Reference
		Black	13.3%	36.5%	21.8%	26.9%	5.10%
		Hispanic	12.0%	30.3%	23.1%	33.1%	10.00%
Potter et al. (2024) [20]	Southern Community Cohort Study	White	59.1%	74.4%	Reference	Reference	Reference
		Black	33.5%	57.4%	25.6%	17.0%	−8.60%
Aldrich et al. (2019) [10]	Southern Community Cohort Study	White	31.0%	-	Reference	-	-
		Black	17.0%	-	14.0%	-	-
Potter et al. (2024) [20]	Southern Community Cohort Study and Black Women’s Health Study	White	74.0%	-	Reference	-	-
		Black	57.6%	-	16.4%	-	-
Ryan et al. (2016) [9]	National Cancer Institute-Maryland Lung Cancer Study	European American	39.4%	-	Reference	-	-
		Black American	34.0%	-	5.4%	-	-
Tailor et al. (2020) [11]	American Community Survey, Behavioral Risk Factor Surveillance System, Tobacco Use Supplement to the Current Population Survey	White	5.4%	-	Reference	-	-
		Black	2.0%	-	3.4%	-	-
		Hispanic	0.7%	-	4.7%	-	-

Five studies examined the United States as a whole, especially those using the BRFSS, while others focused on individual states (*n* = 12) or groups of states (*n* = 9). The most frequent inequality calculations were based on comparing Black Americans with white Americans (*n* = 12). Others used more extensive and refined racial categories. Four compared Black, Hispanic, and those categorized as “Other” with white Americans. One included Chinese, Filipino, Japanese, Korean, White, Native Hawaiian, Other Pacific Islander, Hispanic, and other racial and ethnic groups. A final study compared Black, Japanese, Hispanics, and Native Hawaiian, with White adults.

Most studies analyzed inequalities in lung-cancer screening access (*n* = 17), while the other studies quantified inequalities in eligibility (*n* = 12). The total exceeds 26 because three studies cover both access and eligibility inequalities.

First, we review the studies quantifying inequalities in eligibility, followed by those evaluating access. We disaggregate these latter access studies by geographic region, racial group, and data source to assess variations in the estimated magnitude of absolute inequalities.

### Racial inequalities in eligibility (n = 12)

Of the 12 studies examining LCS eligibility by race, 11 found that Black Americans, Hispanics and Asian Americans had lower eligibility than White Americans. One study investigating American Indians, however, reported that they had higher eligibility [30], and another found higher rates among Native Hawaiians [31]. The magnitude of inequalities varied considerably. Black Americans had an estimated lower eligibility, between 3.4 and 26.9 (ppts), Hispanics between 4.7 and 33.1, and Asian Americans between 8.0 and 10.1 ppts.

Next, we investigated the impact of the 2021 guideline reforms on racial inequalities based on studies reporting both pre- and post-reform eligibility. Eight studies estimated pre-2021 levels of racial inequalities: all studies examined data on Black Americans, six included data on Hispanics, and only two analyzed data on Asian Americans.

Overall, the guideline changes appeared to increase eligibility coverage across all racial and ethnic groups, as shown in Table 1. However, since White populations experienced a similar magnitude of increase in access, and in several cases greater, than minority populations, racial inequalities in access persisted at similar magnitudes over time.

Overall, all eight studies showed an increase in LCS eligibility for Black Americans in association with the 2021 changes. For example, Potter et al. analyzed nationally representative data from the Southern Community Cohort Study, covering the period from 2002 to 2021. They reported that Black Americans had a 33.5% eligibility rate for LCS in 2013, which increased to 57.4% after the 2021 updates [20]. Additionally, Li et al. analyzed 2016 national data from the Health and Retirement Study and found that 13.3% of Black Americans met eligibility for LCS in 2013, rising to 36.5% after 2021 [37]. In parallel, all six studies on Hispanics showed increased LCS eligibility after the changes. For example, Li et al. demonstrated that Hispanics had an increase in eligibility for LCS, improving from 12.0 to 30.3% after the 2021 changes [37]. Moreover, Aredo et al. examined lung cancer cases data from the Multiethnic Cohort from 1993 to 2017 in California and Hawaii [31]. They applied both the 2013 and 2021 guidelines to assess how many individuals diagnosed with lung cancer would have been eligible for screening under each set of criteria. Their analysis showed that Hispanics had a 28.9% eligibility in 2013, which increased to 37.3% with the 2021 guidelines [31]. Similarly, eligibility among Asian Americans increased from 33.4 to 40.0% with the changes [31].

Yet, as shown in Table 1, inequalities persisted and, in several cases, widened. Taking Williams and colleagues’ study as an example in their study of BRFSS data, they estimated that LCS eligibility rose in White Americans from 21.9% in 2013 to 35.8% in 2021, while for Black Americans, this change was from 16.0 to 28.5%, and for Hispanics it was from 9.8 to 18.0%, respectively [32]. This corresponds to a change in absolute inequality for Blacks from 5.9% in 2013 to 7.3% in 2021 and for Hispanics from 12.1 to 17.8% [32]. Thus, despite improvements in eligibility for all populations, in this study, inequalities widened.

### Inequalities in access by geographic location

We next evaluated studies that attempted to compare the magnitude of inequalities across geographic regions. When comparing racial inequality in access, the highest disparity appeared in Pennsylvania. Using the Jane and Leonard Korman Respiratory Institute Lung Cancer Screening Program data, researchers found that access among White Americans was 12.9 ppts higher ppts than among Black Americans [3]. On the other hand, the lowest disparity in access occurred in Georgia, where White Americans had 1.54 ppts higher LCS rates than Black Americans [34]. When comparing the West and East regions of the US, larger inequalities were generally found on the East coast. The greatest racial inequality in access on the East Coast was 12.9 ppts, while on the West Coast, other racial and ethnic minorities had higher access compared to White Americans [3, 38, 39]. For example, Oshiro et al. showed that Korean Americans had 14.0 ppts higher LCS rates than White Americans in Hawaii [38]. Furthermore, a study in California found that Black Americans had 2.5 ppts higher lung cancer screening rates than White Americans [39].

### Racial inequalities in access (n = 17)

The observed racial inequalities in eligibility also corresponded into inequalities in access. However, overall, in the 17 included studies, inequalities appeared to narrow slightly when investigating whether eligible persons actually were able to access to LCS services. See Table 2. Similar to the observations for inequalities in eligibility, Black Americans exhibited the greatest inequality in access when compared with white populations, ranging from between 1.42 and 12.9 ppts.

**Table 2:** Access to lung cancer screening (*n* = 17)

Study	Population Studied	Racial group	Access to Lung Cancer Screening	Absolute Inequality
Navuluri et al. (2023) [16]	Durham Veterans Affairs Health Care System Lung Cancer Screening Database	White	40.3%	Reference
		Black	30.5%	9.9%
Hughes et al. (2023) [34]	County Health Rankings	White	3.71%	Reference
		Black	2.2%	1.5%
Lake et al. (2020) [3]	Jane and Leonard Korman Respiratory Institute Lung Cancer Screening Program	White	76.7%	Reference
		Black	63.8%	12.9%
Oshiro et al. (2022) [38]	Kaiser Permanente Hawaii EMR Data	Korean	94.0%	Reference
		Filipino	79.0%	15.0%
		Japanese	88.0%	6.0%
		Chinese	82.0%	12.0%
		White	80.0%	14.0%
		Native Hawaiian	80.0%	14.0%
		Pacific Islander	79.0%	15.0%
		Hispanics	79.0%	15.0%
Richmond et al. (2020) [17]	Community-based Cancer Center	White	8.0%	Reference
		Black	1.7%	6.3%
Steiling et al. (2020) [40]	Boston Medical Center Lung Cancer Screening Program	White	64.8%	Reference
		Black	55.4%	9.4%
		Asian	90.9%	−26.1%
Wong et al. (2024) [39]	A tertiary care, academic center in Northern California	White	87.5%	Reference
		Black	90.0%	−2.5%
		Asian	84.4%	3.1%
Gudina et al. (2023) [41]	Behavioral Risk Factor Surveillance System	White	16.03%	Reference
		Black	14.61%	1.42%
		Hispanic	16.67%	−0.6%
Japuntich et al. (2018) [25]	Lifespan Medical System	Non-Black	13.0%	Reference
		Black	3.0%	10.0%
Poghosyan et al. (2021) [18]	Behavioral Risk Factor Surveillance System	White	17.4%	Reference
		Black	12.0%	5.4%
Poulson et al. (2022) [35]	Boston Medical Center Lung Cancer Screening Program	White	65.0%	Reference
		Black	55.1%	9.9%
Shin et al. (2022) [36]	A tertiary care, academic center in New England	White	-	Relative
		Black	-	-
		Asian	-	-
Shusted et al. (2023) [42]	Jefferson Lung Cancer Screening Program	White	85.8%	Reference
		Black	73.3%	12.5%
Lozier et al. (2021) [43]	Behavioral Risk Factor Surveillance System	White	15.4%	Reference
		Black	13.2%	2.2%
Narayan et al. (2021) [30]	Behavioral Risk Factor Surveillance System	White	-	Relative
		Black	-	-
		Hispanic	-	-
		Asian	-	-
		American Indian	-	-
Williams et al. (2022) [32]	Behavioral Risk Factor Surveillance System	White	20.8%	Reference
		Black	20.2%	3.6%
		Hispanic	23.8%	−3.0%
Tailor et al. (2020) [11]	Behavioral Risk Factor Surveillance System	White	5.4%	Reference
		Black	2.0%	3.4%
		Hispanic	0.7%	4.7%

Across studies, we found several noteworthy differences in racial inequality patterns, whereby, in some cases, minority groups exhibited greater access than white populations. For example, William et al. analyzed BRFSS data from 2019 across 20 states and found that access among Hispanics was 3 ppts higher than White Americans [32]. Guidna et al. examined the same dataset, but over a timeframe from 2018 to 2020 and across the entire country. Here, Hispanics were found to be 1.85 times more likely to screen for lung cancer compared to White Americans [41]. Another study by Oshiro et al., using data from Kaiser Permanente Hawaii electronic medical records, found that Korean Americans had 14.0% points higher LCS rates than White Americans, 6.0 points higher than Japanese Americans, 12.0 points higher than Chinese Americans, 15.0 points higher than Filipino Americans, 14.0 points higher than Native Hawaiian, 15.0 points higher than other Pacific Islander, and 15.0 points higher than Hispanics [38]. Similarly, a study conducted by Steiling et al. explored data from Boston Medical Center Lung Cancer Screening program [40]. This study found that Asian Americans had a screening rate 26.4 ppts higher than White Americans [40]. Moreover, analyses by Wong et al. and Lozier et al. revealed that Black Americans had LCS rates 2.5 ppts higher than White Americans [39, 43].

Two studies analyzed data on both eligibility and access, reporting inequalities in both absolute and relative terms [11, 30]. In line with previous findings, Tailor et al. showed that White Americans had higher eligibility for LCS by 3.4 ppts compared to Black Americans, 4.7 ppts compared to Hispanics, and 3.2 ppts compared to other racial and ethnic groups [11]. In contrast, a study by Narayan et al. among 20 states using BRFSS data found that although White Americans had higher LCS eligibility than Black Americans, Hispanics, and Asian Americans, there was no evidence of racial differences in actual screening rates for those who were eligible [30]. Specifically, White Americans had higher eligibility for LCS by 6.0 ppts as compared to Black Americans, 10.0 points when compared to Hispanics, and 10.0 ppts as compared to Asian Americans [30]. However, when comparing screening rates among eligible respondents, the odds of LCS were not significantly different between White and Hispanic respondents (AOR = 0.63; 95% CI: 0.24, 1.65; *P* =.35), White and Black respondents (AOR = 0.80; 95% CI: 0.47, 1.38; *P* =.42), White and American Indian respondents (AOR = 0.70; 95% CI: 0.37, 1.33; *P* =.28), or White and Asian or Pacific Islander respondents (AOR = 4.87; 95% CI: 0.67, 35.49; *P* =.12) [30].

## Discussion

Our systematic review highlights several important observations about the evolution of inequalities in eligibility for and access to LCS across the United States. First, consistent with prior studies, we identified that White Americans generally have both significantly higher eligibility and access rates for LCS compared to Black Americans and Hispanics, but not Asian Americans. However, we also found that these inequalities are, to a great extent, structural; that is, the USPSTF guidelines for eligibility appear to generate relative disadvantages for most minority groups. Second, our study found that revisions to eligibility guidelines in 2021, although increasing access for all racial groups, disproportionately benefitted white populations in multiple studies. Overall, it appeared to, at best, result in no change to racial inequalities in eligibility for LCS. Third, our study found that, overall, inequalities in eligibility were greater than the magnitude of inequalities in actual access among eligible persons. Finally, we found evidence that these inequalities were not inevitable: the East Coast region of the US exhibited considerably higher inequalities in access than in the West Coast. In particular, California had greater access among black populations, which could reflect a greater targeted allocation to this group.

Before further interpreting our findings, we must note some important limitations. First, the scope of this study was limited to a systematic review rather than an original data analysis. We chose this approach because, to our knowledge, no prior review has synthesized the available evidence on racial disparities in LCS eligibility and access in the United States. A systematic review enabled us to identify patterns and trends across multiple studies that would not have been possible through smaller analyses. In our review, we had to rely on either small longitudinal samples or repeated cross-sectional data, neither of which allowed us to draw robust causal inferences. Future research should explore these disparities by conducting a natural experiment or longitudinal design to understand the impact of the changes in guidelines.

Next, as we focused on the US, our findings are not generalizable to the state of racial inequalities in screening in other countries. Furthermore, we only included peer-reviewed articles, and there may not any grey literature. Additionally, we cannot exclude the risk of publication bias against studies reporting negative findings. Both of these limitations could be addressed in future work through an examination of gray literature and government reports, which may be more likely to publish negative findings. Moreover, the substantial heterogeneity across study designs, population denominators, and inequality measures made a meta-analysis technically infeasible, thereby constraining our ability to generate pooled effect estimates and limiting our quantitative synthesis [44].

Several limitations also arose from the included studies themselves. Many studies lacked standardized measures for assessing eligibility and access, leading to inconsistencies in how these outcomes were reported and limiting comparability and generalizability. For example, population denominators were different for each study. There were seven studies that used the BRFSS data, but the study period and populations were different. Furthermore, some studies focused on individuals eligible for LCS, while others included only ever-smokers or individuals associated with Medicare to analyze the population data. Another limitation concerns our search strategy. Although our protocol-specified search did not include MeSH terms, we conducted a supplemental MeSH-inclusive search and confirmed that it produced the same set of included studies. Given that our review was pre-registered with PROSPERO, we adhered to the original strategy to maintain consistency with the protocol. Importantly, the additional search confirmed that the omission of MeSH terms did not alter the set of included articles, supporting the robustness of our findings. Additionally, our review was limited to peer-reviewed publications, which may have introduced linguistic and publication bias. The exclusion may have resulted in the omission of gray literature reporting negative results. Moreover, the reliance on observational studies across the studies in our literature limits the ability to make causal inferences since many of these studies did not adjust for potential confounders. Finally, the limited representation of Asian American populations in most of the studies raises concerns about the generalizability of findings for this race.

Notwithstanding these limitations, this review provides valuable insights into the current state of research on racial inequality in LCS access. To our knowledge, this is the first systematic review specifically addressing this issue, and we have shown that inequalities in LCS access and eligibility are pronounced and complex. The strengths of our review include a comprehensive search strategy, a clear application of PRISMA guidelines, and a systematic approach to data extraction that captured a wide array of study characteristics. Furthermore, our focus on multiple racial and ethnic groups, as well as geographic differences, enrich our understanding of how inequalities manifest across different populations.

One noteworthy observation is that inequalities in access persisted, or even widened, despite overall improvements in eligibility across all racial groups. This is consistent with other health equity literature finding that public health interventions can sometimes increase health inequalities. This finding also demonstrates the persistence of the ‘inverse care law’ in LCS services in the US. While it is often speculated that the inverse care law is mostly a result of market forces in care [5], here we find that, at least in part, it can stem from institutional factors, specifically screening guidelines. Geographic disparities may also reflect differences in state-level Medicaid expansion, public health system infrastructure, and local outreach strategies. These factors have been shown to influence cancer screening uptake across the United States. Our systematic review found that West Coast states had higher LCS rates than East Coast states [45, 46]. This may be explained by vigorous Medicaid policies, such as California’s early and expansive adoption of Affordable Care Act (ACA) provisions and Medicaid managed care programs that highly promote preventive services among low-income and minority populations [47]. Furthermore, other reasons contributing to the regional variation may include but are not limited to structural differences in healthcare infrastructure, insurance coverage, and socioeconomic status [48, 49]. A review of the ACA also supports this rationale by demonstrating that states that expanded Medicaid had a greater increase in cancer screening rates than non-expansion states [47].

However, not all populations fit the broader trend of decreased access among racial and ethnic minorities. For example, in Hawaii, Korean Americans demonstrated higher LCS rates than both white Americans and other Asian subgroups. Korean Americans’ higher screening uptake may reflect the alignment of cultural perceptions – such as strong preventative care and family involvement – with healthcare delivery models that offer linguistically and culturally tailored services. For example, Kaiser Permanente Hawaii implements Korean-language outreach, culturally tailored health education, and community-based cultural interventions to reduce barriers and promote cancer screening among Korean Americans [50]. These culturally tailored services may surpass those routinely available to White populations, especially when collaborated with strong community trust and engagement for Korean Americans. In contrast, Chinese, Filipino, and other Asian American subgroups may experience greater variation in acculturation, language barriers, or health literacy, which may limit their barriers and facilitators to their screening participation. The screening rate differences among Asian subgroups highlight the importance of disaggregating data and tailoring interventions to each community’s unique cultural, linguistic, and structural contexts.

Our study points to several important directions for future research and practice. First, there is a critical need for further standardization in how eligibility and access to LCS are defined and measured, as this variability impedes the reproducibility and comparability of results across studies. Future research should also further investigate the underlying factors contributing to inequalities in access, such as socioeconomic status and healthcare provider biases which might help explain for instance, higher eligibility and access rates for Asian Americans [51, 52]. This can also help inform the development of tailored interventions to improve LCS access for underserved populations. Future work is also needed to examine what sort of changes in the USPSTF screening guidelines might prove more beneficial to ameliorating racial inequalities; one possibility is that they should take other important risk factors into consideration, such as genetic predisposition [53–55] and disproportionate exposure among minority groups to harmful exposures such as neighborhood disadvantage, air pollution, and second-hand smoke [56, 57]. Recent studies have explored the potential of incorporating polygenic risk scores (PRS) into screening criteria to improve individualized lung cancer risk assessment [58–60]. For example, Trendwoski et al. found that a PRS was predictive of lung cancer risk among White individuals but failed to show predictive power among Black participants [58]. This highlighted the need for ancestry-specific validation.

## Conclusion

Taken together, our research demonstrates that despite policy initiatives to curb inequalities, significant racial inequalities persist in LCS eligibility and access across the US. Following the 2021 guideline changes intended to reduce racial inequalities, our review conducted in 2025 failed to identify clear improvements in eligibility disparities —particularly among Black and Hispanic populations — although the available evidence was limited. Continued research and intervention in this area can help foster more equitable access to LCS and, ultimately, reduce avoidable inequalities in mortality rates associated with lung cancer.

## Supplementary Information

Supplementary Material 1.

Supplementary Material 2.

## Data Availability

The datasets used and analyzed during the current study are available from the corresponding author upon reasonable request. Here is a link to the Dropbox of the included studies: https://www.dropbox.com/scl/fo/n5hqxqy2469gsgbx2bbup/ABzhHc1HgiZ9IcgrprroUlw?rlkey=0q2yogi7g0euduwxfv0oyvog8&e=1&st=ea7mt2gp&dl=0.

## Appendix

### Verbatim search strings

#### PubMed

##### 589 Results

("race"[Title/Abstract] OR "people of colo*"[Title/Abstract] OR "minori*"[Title/Abstract] OR "ethnic*"[Title/Abstract] OR "immigrant*"[Title/Abstract] OR "emigrant"[Title/Abstract] OR "emigrat*"[Title/Abstract] OR "African American"[Title/Abstract] OR "African-Americans"[Title/Abstract]) AND ("Lung cancer"[Title/Abstract] OR "lung disease"[Title/Abstract] OR "lung neoplasm"[Title/Abstract]) AND ("Screening"[Title/Abstract] OR "low-dose computed tomography"[Title/Abstract] OR "LDCT"[Title/Abstract])

#### Web of Science

##### 896 Results

TS=(race OR “people of colo*” OR minori* OR ethnic* OR immigrant* OR emigrant OR emigrat* OR “African American” OR “African-Americans”)TS=(“Lung cancer” OR “lung disease” OR “lung neoplasm”)TS=(Screening OR “low-dose computed tomography” OR LDCT)#1 AND #2 AND #3

## Abbreviations

LCS: Lung cancer screening
USPSTF: U.S. Preventative Services Task Force
BRFSS: Behavioral Risk Factor Surveillance System
PRISMA: Preferred Reporting Items for Systematic Reviews and Meta-Analyses
