# Supplementary material for: Racial inequalities in eligibility and access to lung cancer screening: systematic review of United States studies
Source: BMC Public Health. 2025 Nov 17;25:3978. doi: 10.1186/s12889-025-24761-2 (PMC12625044; doi:10.1186/s12889-025-24761-2)
Supplement: Supplementary file 1 — Supplementary Material 1. [file 12889_2025_24761_MOESM1_ESM.docx]

Appendix 2. Eligibility Studies Data Extraction Table

| **Study** | **Year** | **Location** | **Population** | **Study Period** | **Data Source** | **Racial Comparison** | **Access Outcome** | **Inequality Measure** | **Results** |
| --- | --- | --- | --- | --- | --- | --- | --- | --- | --- |
| Ryan et al. | 2016 | Maryland | Individuals with lung cancer | 1998-2014 | National Cancer Institute-Maryland Lung Cancer Study | Black American, European American | Eligibility | Absolute Inequality | 2013: European Americans had 5.4 higher percentage points eligibility for lung cancer screening than Black Americans. |
| Aldrich et al. | 2019 | 12 states | Individuals eligible for lung cancer screening | 2014-2022 | Southern Community Cohort Study | White, Black | Eligibility | Absolute Inequality | 2013: White Americans had 14.0 higher percentage points eligibility for lung cancer screening than Black Americans. |
| Tailor et al. | 2020 | USA | Medicare beneficiaries | 2012-2016 | American Community Survey, Behavioral Risk Factor Surveillance System, Tobacco Use Supplement to the Current Population Survey | White, Black, Hispanic, Other | Eligibility and Access | Absolute and Relative Inequality | White Americans had 3.4 percentage points higher eligibility for lung cancer screening than Black Americans and 4.7 points higher than Hispanics.  Compared to White Americans, Black Americans were 2.27 times and Hispanics were 6.67 times less likely to screen for lung cancer |
| Reese et al. | 2021 | 19 states | Ever smokers | 2017-2018 | Behavioral Risk Factor Surveillance System | White, Black, Hispanic | Eligibility | Absolute Inequality | 2013: White Americans had 14.8 percentage points higher eligibility for lung cancer screening than Black Americans and 20.6 points higher than Hispanics.  2021: White Americans had 12.1 percentage points higher eligibility for lung cancer screening than Black Americans and 22.2 points higher than Hispanics. |
| Narayan et al. | 2021 | 20 states | Ever smokers | 2019 | Behavioral Risk Factor Surveillance System | White, Black, Hispanic, Asian, American Indian, Other | Eligibility and Access | Absolute and Relative Inequality | 2013 (Eligibility): White Americans had 5.0 higher percentage points eligibility for lung cancer screening than Black Americans, 8.0 points higher than Hispanics, 8.0 points higher than Asian Americans or Pacific Islanders, but 5.0 points lower than American Indians.   2013 (Eligibility in relative inequality): Compared with White Americans, Black Americans were 2.78 times less likely to be eligible for lung cancer screening and Hispanics were 6.67 times less likely. There was no evidence differences comparing eligibility in White Americans versus Asians, and American Indians.  2021 (Eligibility): White Americans had 6.0 higher percentage points eligibility for lung cancer screening than Black Americans, 10.0 points higher than Hispanics, 10.0 points higher than Asian Americans or Pacific Islanders, but 6.0 points lower than American Indians.  2021 (Eligibility in relative inequality): Compared with White Americans, Black Americans were 2.56 times less likely to be eligible for lung cancer screening and Hispanics were 6.67 times less likely. There was no evidence differences comparing eligibility in White Americans versus Asians and American Indians.  Compared with White Americans, there was no evidence of differences in getting lung cancer screening among Black Americans, Hispanics, Asians, and American Indians. |
| Aredo et al. | 2022 | California and Hawaii | Ever-smoking lung cancer cases | 1993-2017 | Multiethnic Cohort | Black, Japanese American, Hispanics, Native Hawaiian, White | Eligibility | Absolute Inequality | 2013: White Americans had 15.8 higher percentage points eligibility for lung cancer screening than Black Americans, 10.1 points higher than Japanse Americans, 14.6 points higher than Hispanics, but 2.8 points lower than Native Hawaiian.  2021: White Americans had 11.2 higher percentage points eligibility for lung cancer screening than Black Americans, 9.6 points higher than Japanse Americans, 12.3 points higher than Hispanics, but 7.1 points lower than Native Hawaiian. |
| Pinheiro et al. | 2022 | 48 states and D.C. | Ever smokers | 2003-2007 | REGARDS study | White, Black | Eligibility | Absolute Inequality | 2013: White Americans had 8.0 higher percentage points eligibility for lung cancer screening than Black Americans.  2021: White Americans had 5.8 higher percentage points eligibility for lung cancer screening than Black Americans. |
| Williams et al. | 2022 | 20 states | Ever smokers | 2019 | Behavioral Risk Factor Surveillance System | White, Black, Hispanic, Other | Eligibility and Access | Absolute Inequality | 2013 (Eligibility): White Americans had 5.9 higher percentage points eligibility lung cancer screening rates than Black Americans and 12.1 percentage points higher than Hispanics.  2021 (Eligibility): White Americans had 7.3 higher percentage points eligibility lung cancer screening rates than Black Americans and 17.8 percentage points higher than Hispanics.  Hispanics had 3.0 percentage points higher lung cancer screening rates than White Americans and 3.6 points higher than Black Americans. |
| Liu et al. | 2023 | Massachusetts | Individuals with lung cancer | 2015-2020 | Boston Medical Center’s Clinical Data Warehouse | White, Black, Hispanic, Other | Eligibility | Absolute Inequality | 2013: White Americans had 16.3 percentage points higher eligibility for lung cancer screening than Black Americans and 1.3 percentage points higher than Hispanics.  2021: White Americans had 8.1 percentage points higher eligibility for lung cancer screening than Black Americans and 2.4 percentage points higher than Hispanics. |
| Potter et al. | 2024 | USA | Ever smokers | 1995-2021 | Southern Community Cohort Study and Black Women’s Health Study | White, Black | Eligibility | Absolute Inequality | 2021: White Americans had 16.4 higher percentage points eligibility for lung cancer screening than Black Americans. |
| Li et al. | 2024 | USA | Ever smokers | 2016 | Health and Retirement Study | White, Black, Hispanic, Other | Eligibility | Absolute Inequality | 2013: White Americans had 21.8 percentage points higher eligibility for lung cancer screening than Black Americans and 23.1 points higher than Hispanics.  2021: White Americans had 26.9 percentage points higher eligibility for lung cancer screening than Black Americans and 33.1 points higher than Hispanics. |
| Potter et al. | 2024 | 12 | Individuals with lung cancer | 2002-2021 | Southern Community Cohort Study | White, Black | Eligibility | Absolute Inequality | 2013: White Americans had 25.6 higher percentage points eligibility for lung cancer screening than Black Americans.  2021: White Americans had 17.0 higher percentage points eligibility for lung cancer screening than Black Americans. |
