# Supplementary material for: Racial inequalities in eligibility and access to lung cancer screening: systematic review of United States studies
Source: BMC Public Health. 2025 Nov 17;25:3978. doi: 10.1186/s12889-025-24761-2 (PMC12625044; doi:10.1186/s12889-025-24761-2)
Supplement: Supplementary file 2 — Supplementary Material 2. [file 12889_2025_24761_MOESM2_ESM.docx]

Appendix 1. Verbatim search strings.

PubMed

**589 Results**

("race"[Title/Abstract] OR "people of colo*"[Title/Abstract] OR "minori*"[Title/Abstract] OR "ethnic*"[Title/Abstract] OR "immigrant*"[Title/Abstract] OR "emigrant"[Title/Abstract] OR "emigrat*"[Title/Abstract] OR "African American"[Title/Abstract] OR "African-Americans"[Title/Abstract]) AND ("Lung cancer"[Title/Abstract] OR "lung disease"[Title/Abstract] OR "lung neoplasm"[Title/Abstract]) AND ("Screening"[Title/Abstract] OR "low-dose computed tomography"[Title/Abstract] OR "LDCT"[Title/Abstract])

Web of Science

**896 Results**

TS=(race OR “people of colo*” OR minori* OR ethnic* OR immigrant* OR emigrant OR emigrat* OR “African American” OR “African-Americans” )

TS=(“Lung cancer” OR “lung disease” OR “lung neoplasm” )

TS=(Screening OR “low-dose computed tomography” OR LDCT)

#1 AND #2 AND #3
